# Hepatic lipid droplet breakdown through lipolysis during hibernation in Chinese Soft-Shelled Turtle (*Pelodiscus sinensis*)

**DOI:** 10.18632/aging.101887

**Published:** 2019-03-29

**Authors:** Yufei Huang, Hong Chen, Ping Yang, Xuebing Bai, Yonghong Shi, Waseem Ali Vistro, Imran Tarique, Abdul Haseeb, Qiusheng Chen

**Affiliations:** 1 MOE Joint International Research Laboratory of Animal Health and Food Safety, College of Veterinary Medicine, Nanjing Agricultural University, Nanjing, Jiangsu Province 210095, China; 2 Shanghai Veterinary Research Institute, Chinese Academy of Agricultural Sciences, Shanghai 200241, China

**Keywords:** hibernation, Chinese soft-shelled turtle, lipid droplet, hydrolysis, ATGL

## Abstract

Hibernation is an adaptive survival strategy in response to cold and foodless winter. To determine the underlying mechanisms of seasonal adaptions, transcriptome sequencing studies have been conducted in bears, ground squirrels and bats. Despite advances in identifying differentially expressed genes involved in metabolism, the precise mechanisms of these physiological adaptions remain unclear. In the present study, we examined liver of Chinese Soft-Shelled Turtle (*Pelodiscus sinensis*) and found that the contents of lipid droplet (LD) and triglyceride (TG) were significantly decreased during hibernation. Increases in mRNA expression levels of lipolysis-related genes and decreased levels of lipogenesis-related genes during hibernation indicated that LD hydrolysis was stimulated during hibernation. To continuously release fatty acids (FAs) from LD, adipose triglyceride lipase (ATGL) was recruited and accumulated on the surface of LDs via activation of Cyclic Adenosine monophosphate (cAMP)/protein kinase A (PKA) signaling. Meanwhile, increased phosphorylation of the LD-associated protein, perilipin-5, activated the enzyme activity of ATGL via interaction between comparative gene identification-58 (CGI-58) and ATGL. Taken together, our results indicated that ATGL accumulation on the LD surface and its induced enzyme activity during hibernation promoted LD breakdown in the liver of Chinese Soft-Shelled Turtle (*Pelodiscus sinensis*), thereby enhancing mitochondrial β-oxidation to maintain energy hemostasis.

## Introduction

With increased average life expectancy over the past few decades, the global population is aging fast [1]. Meanwhile, the structure or function of physiological systems is slowing, progressively changing and deteriorating in the aging process. The effects of aging on structure and function of body and liver promote the occurrence of chronic liver disease, especially nonalcoholic fatty liver disease (NAFLD) among the elderly [2]. Histologically, the characteristic feature of NAFLD is liver fat deposition. Despite numerous researches in NAFLD, few effective approaches to degrade hepatic lipid droplet (LD) have been found.

Hibernation is an energy-limited period, during which organisms lower their metabolic rate to adapt to a colder winter or food restriction. The Chinese Soft-Shelled Turtle (*Pelodiscus sinensis*) usually started to hibernate in the middle of November and come out of hibernation in the middle of April [3,4]. Before hibernation, the amount of triglyceride (TG) stored as LD in the liver of hibernating animals is significantly increased [5]. On entering hibernation, LD is gradually degraded [6]. Therefore, large amounts of lipids that accumulated in the non-hibernation period are gradually metabolized during hibernation [7]. This interesting phenomenon inspired us that understanding the molecular mechanism of hepatic LD breakdown in Chinese Soft-Shelled Turtle (*Pelodiscus sinensis*) during hibernation may therefore provide valuable insight towards a new therapy target for NAFLD.

LDs are main storage sites of cellular neutral lipids and widely existed in bacteria, yeast, plants, insects, and animal cells [8]. The biological functions of LDs did not attract researchers’ attention until the discovery of lipid hydrolases [9] and lipid droplet structural proteins [10–14]. In the face of increasing energy demands by the body, TG stored in cellular LDs will be broken down via enzyme-catalyzed lipolysis. In this process, ATGL first hydrolyzes TG into diacylglycerol (DG) and DG is then decomposed to monoacylglycerol (MG) by hormone sensitive lipase (HSL). Ultimately, MG is catalytically hydrolyzed into glycerol by monoacylglycerol lipase (MGL). The fatty acids (FAs) produced at each hydrolytic step and glycerol are important sources of energy for the body. Furthermore, LD-related proteins have been proven to be involved in enzyme catalyzed lipolysis. In adipose tissues, phosphorylation of perilipin-1 via β-adrenergic activation of PKA increases ATGL activity [15,16]. Nevertheless, in oxidative tissues, such as the liver and skeletal muscle, perilipin-5, rather than perilipin-1, directly anchors ATGL and its activator (CGI-58 also known as comparative gene identification-58) to improve the enzymatic activity [17]. Although the roles of lipid hydrolases and LD proteins related to LD decomposition have been widely studied *in vivo* and *in vitro*, the main experimental results were obtained using common animal models, such as mouse and rat. However, few studies in this field have been conducted to study its role in hibernating animals, let alone amphibians and reptiles.

To identify genes responsible for energy metabolism during hibernation, transcriptome sequencing studies have been conducted in hibernating animals. In the liver of American black bear (*Ursus americanus*), a 12,800 cDNA probe microarray experiment showed that genes involved in carbohydrate synthesis and fatty acid β-oxidation were up-regulated, while genes responsible for carbohydrate catabolism and lipid biosynthesis were down-regulated [18]. Transcriptome sequencing in the liver of Greater Horseshoe Bat (*Rhinolophus ferrumequinum*) [19] and thirteen-lined ground squirrel (*Spermophilus tridecemlineatus*) [20], also detected differentially expressed genes associated with metabolic depression and shifts in fuel utilization. Despite advances in identifying differentially expressed genes responsible for the seasonal accommodation, the specific molecular mechanisms underlying LD hydrolysis in the liver of hibernating animals remain unclear. Therefore, in the present study, we aimed to study the dynamic mechanism responsible for LD breakdown during hibernation in the liver of the Chinese Soft-Shelled Turtle (*Pelodiscus sinensis*).

## RESULTS

### Changes in LD and TG contents in the liver during hibernation

**Figure 1:**
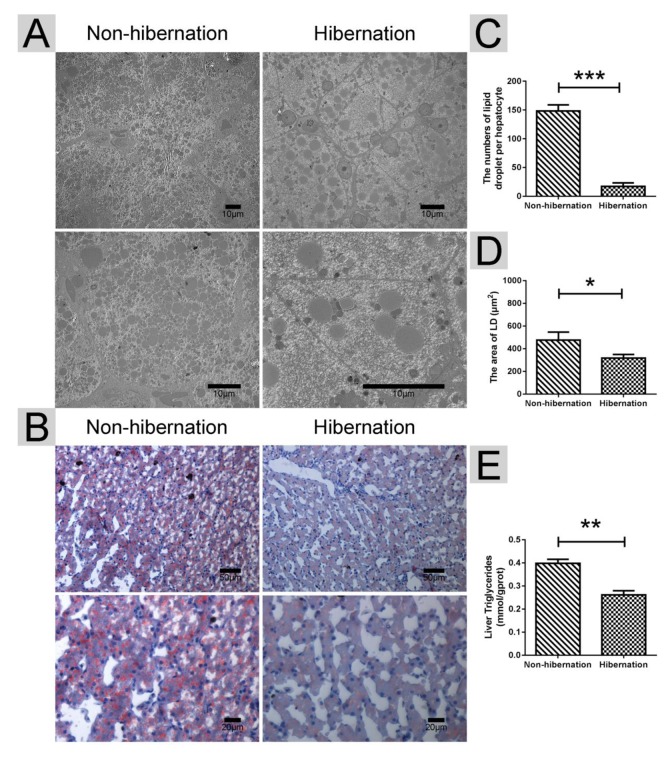
**Differences in the lipid droplet (LD) and triglyceride (TG) contents in the liver during hibernation and non-hibernation group.** (**A**) Transmission electron microscopy (TEM) image of hepatocytes; (**B**) Oil Red O staining of the liver; (**C**) Statistics of LD numbers in the hepatic TEM image; (**D**) Statistics of LD areas; (**E**) Analysis of TG in the liver.

Under TEM, we observed that the numbers of LDs per hepatocyte in the hibernation group were much lower than those in the non-hibernation group (Figure 1A, C). Oil red O staining further indicated the significant decrease in LD contents under light microscopy (Figure 1B, D). Additionally, the hepatic TG contents were dramatically decreased in the hibernation group compared with those in the non-hibernation group (Figure 1E). These results showed that the hepatic LD and TG contents in the liver of the Chinese soft-shelled turtle were decreased during hibernation.

### Lipolysis-stimulating effects were enhanced during hibernation

**Figure 2:**
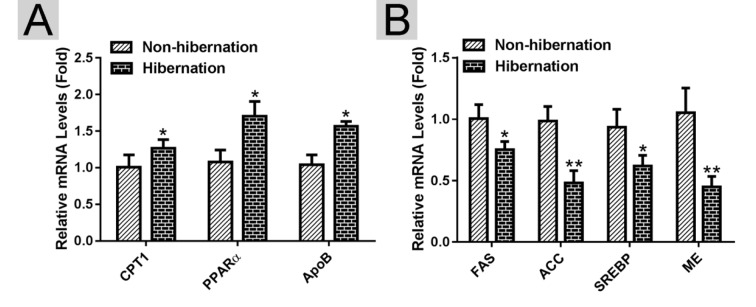
**Identification of differentially expressed genes in the liver of Chinese Soft-Shelled Turtle (*Pelodiscus sinensis*) between hibernation and non-hibernation group.** (**A**) The mRNA expression of lipolysis-related genes (*CPT1*, *PPARα* and *APOB*); (**B**) The mRNA level of lipogenesis-related genes (*FAS*, *ACC*, *SREBP* and *ME*).

**Figure 3:**
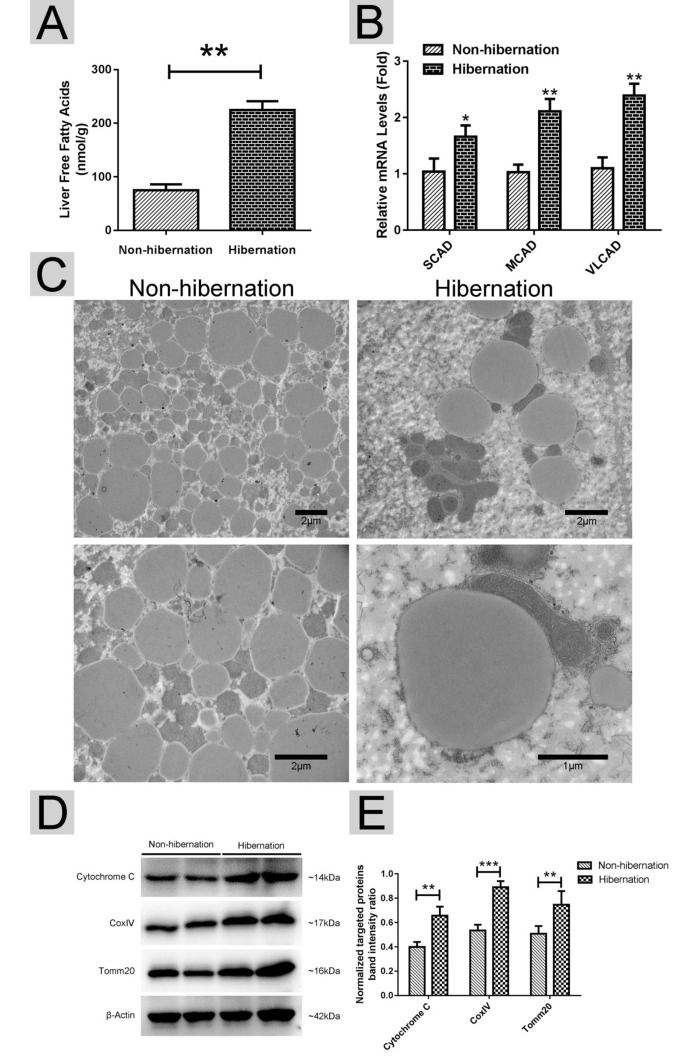
**Lipolysis was stimulated during hibernation.** (**A**) Contents of non-esterified free fatty acids (NEFAs) in the liver; (**B**) The mRNA expression of regulatory enzymes for mitochondrial β-oxidation (SCAD, MCAD and VLCAD); (**C**) Transmission electron microscopy (TEM) image of hepatocytes; (**D**) Western blotting analysis of mitochondrial markers (Cytochrome C, Cox IV and Tomm20); (**E**) Statistics of the western blotting results.

Using RT-PCR, we found that the mRNA expression levels of lipolysis-related genes (carnitine palmitoyltransferase 1A (*CPT1*), peroxisome proliferator activated receptor alpha (*PPARα*), and apolipoprotein B (*APOB*)) were increased, whereas the mRNA expression levels of lipogenesis-related genes (fatty acid synthase (*FAS*), acetyl CoA carboxylase (*ACC*), sterol regulator element binding protein (*SREBP*) and malic enzyme (ME)) were decreased in the hibernation group compared with those in the non-hibernation group (Figure 2). This indicated that lipolysis is up-regulated and lipogenesis is suppressed in the liver of Chinese soft-shelled turtle during hibernation. During lipolysis, cytoplasmic lipid hydrolases will remove FAs from LDs, promoting their access to mitochondria for cellular energy balance. Compared with the non-hibernation group, the contents of non-esterified free fatty acids (NEFAs) in the liver were significantly increased in the hibernation group (Figure 3A). Meanwhile, the mRNA levels of regulatory enzymes for mitochondrial β-oxidation, such as short-chain acyl-CoA dehydrogenase (SCAD), medium-chain acyl-CoA dehydrogenase (MCAD), and very long-chain acyl-CoA dehydrogenase (VLCAD), were elevated in the hibernation group compared with those in the non-hibernation group (Figure 3B). Under TEM, we observed that most mitochondria were circular or oval and maintained a certain distance from LDs in the non-hibernation group (Figure 3C). By contrast, in the hibernation group, the shapes of partial mitochondria were transformed from round or oval to a crescent shape and the mitochondria were tightly associated with the LDs (Figure 3C). Similarly, western blotting analysis showed that the protein levels of mitochondrial markers, including Cytochrome C, Cox IV and Tomm20, were up-regulated in the hibernation group compared with those in the non-hibernation group (Figure 3D, E). This indicated that mitochondrial proliferation was promoted during hibernation. Collectively, these results suggested that lipolysis-stimulating effects in the liver of Chinese soft-shelled turtle were increased during hibernation.

### ATGL accumulation on the surface of hepatic LDs was increased during hibernation

**Figure 4:**
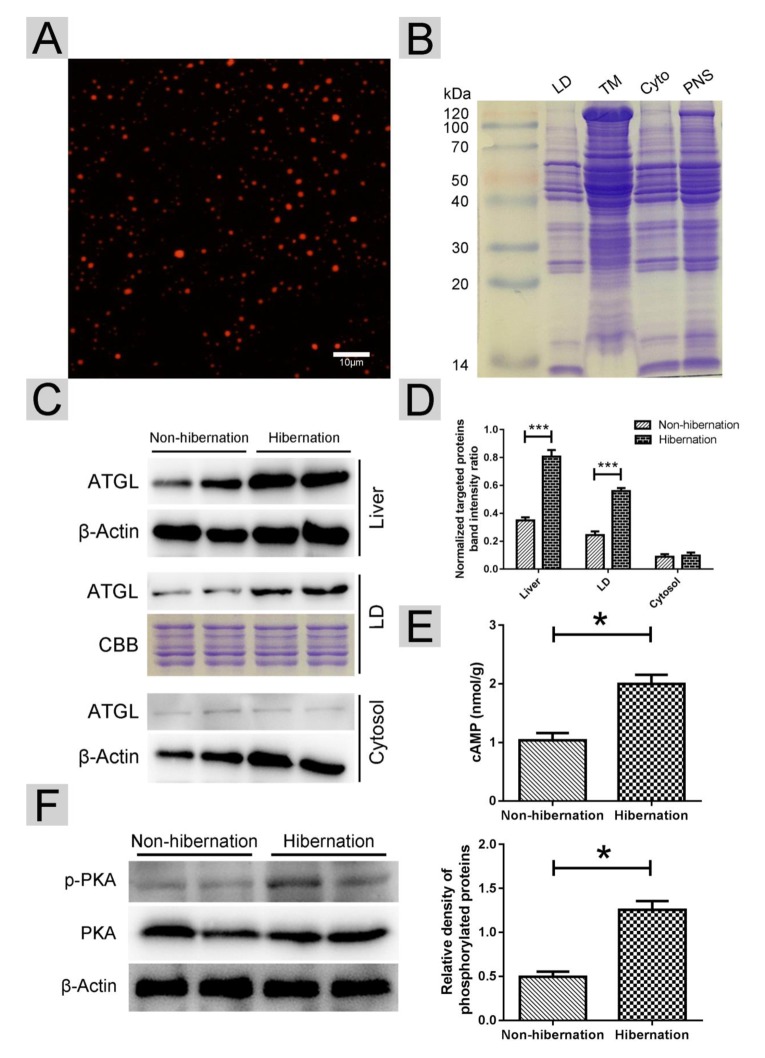
**Activated cAMP/PKA signal increased ATGL accumulation on the surface of LDs.** (**A**) Nile red staining of isolated LDs; (**B**) Colloidal blue staining of LDs, total membrane (TM), cytosol (Cyto) and post-nuclear supernatant (PNS) in the liver; (**C**) Western blot analysis of ATGL protein expression in the liver, LDs and cytosol; (**D**) Statistics of ATGL protein expression; (**E**) The cAMP level in the liver; (**F**) Differential phosphorylation of PKA in the liver.

In lipolysis, ATGL dominates the initial hydrolytic process, releasing FAs and DGs from TGs [21]. ATGL recruitment onto the surface of LDs promotes LD degradation and TG hydrolysis [22]. First, we isolated LDs from livers as previously described [23]. The purity of the LDs was confirmed using Nile red and Colloidal blue staining (Figure 4A, B). Compared with that in the non-hibernation group, the total content of ATGL in the liver and the content in the LDs were increased in the hibernation group. However, the level of ATGL in the cytosol was low and did not change during hibernation (Figure 4C, D). As a main regulator of ATGL [24], the cAMP level was elevated in the hibernation group compared with that in the non-hibernation group (Figure 4E). Likewise, PKA activity was significantly increased during hibernation (Figure 4F). These data illustrated that cAMP/PKA signaling in the liver was activated during hibernation and that ATGL was accumulated on the surface of LDs.

### Perilipin-5 phosphorylation and the interaction of perilipin-5 with ATGL and CGI-58 on hepatic LDs induced ATGL enzyme activity during hibernation

**Figure 5:**
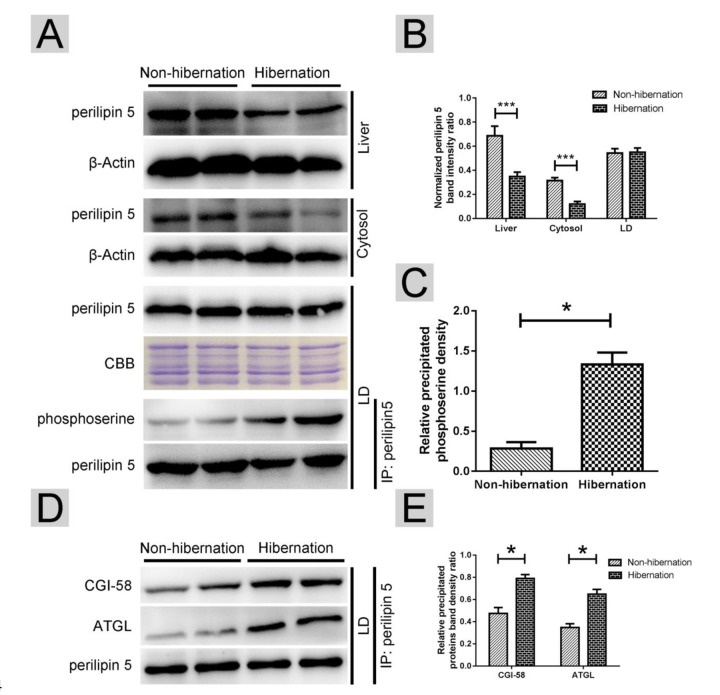
**Phosphorylation of perilipin-5 on LD induced CGI-58-mediated regulation of ATGL activity.** (**A**) Western blotting analysis of perilipin 5 protein expression and perilipin 5 phosphorylation; (**B**) Statistics of perilipin 5 protein expression in the liver, LDs and cytosol; (**C**) Statistics of perilipin 5 phosphorylation in LDs; (**D**) Analysis of the CGI-58/ATGL-perilipin 5 interaction; (**E**) Statistics of precipitated proteins.

In oxidative tissues, such as the liver and skeletal muscle, perilipin-5 is expressed on LDs and participates in the regulation of ATGL [21]. Western blotting results revealed that during hibernation, the total perilipin-5 expression in the liver and in the cytosol decreased (Figure 5A, B). However, the protein expression of perilipin-5 on LDs remained constant during hibernation (Figure 5A, B). Through Co-IP, we discovered that the phosphorylation of perilipin5 on LD surface was increased during hibernation (Figure 5A, C). Previous studies [25] indicated that CGI-58 is an activator of ATGL and could stimulate ATGL by forming a ternary complex of perilipin-5, ATGL, and CGI-58 during fasting. Compared with that in the non-hibernation group, the interaction of perilipin-5 with ATGL and CGI-58 increased in the hibernation group (Figure 5D, E). These results suggested that phosphorylation of perilipin-5 and the interaction of perilipin-5 with ATGL and CGI-58 on the LDs were elevated and induced ATGL enzyme activity during hibernation.

## DISCUSSION

When they encounter a cold and food-limited winter, hibernating animals will drastically reduce their body temperature, heart rate, metabolic rate, and other physiological functions to minimize energy expenditure [26]. Prior to hibernation, hibernating animals rapidly gain weight and increase lipid contents through improving food intake [27]. During hibernation, stored lipids replace carbohydrate as the major energy source [19]. Meanwhile, various aspects of hibernating animals are found to be involved in the hibernation process. After several month of inactivity during hibernation, skeletal muscles of hibernating animals do not dramatically atrophy like non-hibernating animals in spite of partially muscle loss [28]. The discovery of resistance to muscle atrophy in hibernating animals would promote researches in age-related disuse muscle atrophy. Meanwhile, bone loss does not occur in black bear (*Ursus americanus*) and grizzly bear (*Ursus arctos horribilis*) during hibernation [29]. Using brown bears (*Ursus arctos*), the researchers found that winter bear serum could reduce the protein turnover of myotubes, thereby maintain muscle mass and strength during hibernation, and it might be useful for developing new tools to fighting human muscle atrophy [30]. In 13-lined ground squirrels (*Ictidomys tridecemlineatus*) [31], thrombosis and myocardial ischemia do not occur during hibernation (during which non-hibernating animals are more likely to get thrombosis and myocardial ischemia). This phenomenon indicates that hibernating mammalian animals are natural cardiovascular disease research models. As for white adipose tissues (WAT), the main lipids storage organ, hibernating animals storage excessive energy in WAT in active phase and count on WAT degradation during hibernation [6]. Besides, another brown bear paper in subcutaneous adipose tissue showed that increased expression of negative regulators of lipolysis could promote weight gain in summer [27]. As a thermogenic organ for maintaining body temperature, brown adipose tissue (BAT) has been found in the hibernators, such as thirteen-lined ground squirrel (*Ictidomys tridecemlineatus*) [32] and been implicated in therapy for obesity and type 2 diabetes [33]. However, it hasn’t been studied in Chinese Soft-Shelled Turtle (*Pelodiscus sinensis*). In free-ranging brown bears (*Ursus arctos*), the gut microbiota in summer have increased diversity and promote energy storage than hibernation, thus reveals a new treatment for obesity and insulin resistance in age-related diseases [34]. These discoveries in hibernating animals indicate that they could be a potential animal model for age-related diseases (severe muscle atrophy, bone loss, thrombosis, myocardial ischemia, obesity and insulin resistance).

As a vital metabolic organ, liver could preserve excessive energy as TG in LDs and release free fatty acids in response to energy shortages. In the present study, the LD contents in hepatocytes and the hepatic TG contents were decreased, while the contents of NEFAs were significantly increased during hibernation in Chinese soft-shelled turtle. In hepatocytes, the contents of free fatty acids released from TGs are regulated by lipogenesis- and lipolysis-related genes. Consistent with these observations, in the liver of the Chinese Soft-Shelled Turtle, the mRNA expression levels of lipolysis-related genes (*CPT1*, *PPARα* and *ApoB*) increased and lipogenesis-related genes’ (*FAS*, *ACC*, *SREBP* and *ME*) mRNA expression levels decreased during hibernation. To identify genes involved in this metabolic adjustment in the liver during hibernation, transcriptome studies have been carried on mammalian hibernators, for instance, American black bears [18], greater horseshoe bats [19] and ground squirrels [20]. The RNA-sequencing results indicated that genes involved in carbohydrate catabolism and lipid biosynthesis were down-regulated during hibernation, while genes responsible for lipid β-oxidation and protein biosynthesis were up-regulated. These differentially expressed genes demonstrated that the dominating energy metabolic pathways during hibernation switched from carbohydrate oxidation to β-oxidation of released fatty acids. Despite advances in identifying the hepatic genes responsible for metabolic transition, the molecular mechanism and signaling pathway underlying LD decomposition in hibernating animals remains unclear.

As the major hydrolytic enzyme responsible for TG, the regulation of ATGL expression and the consequent activation of its hydrolase activity are complex [35]. A serious of studies demonstrated that *ATGL* mRNA expression is increased by starvation [36], whereas food intake [37] decreases its expression. In addition, insulin [38], isoproterenol [39], mammalian target of rapamycin (mTOR) complex 1 [40], and tumor necrosis factor alpha (TNF-α) [39] could reduce the *ATGL* mRNA levels. Conversely, the mRNA and protein expression of *ATGL* are elevated by catecholamine [41], dexamethasone [36] and the activation of forkhead box protein O1 (FoxO1) [42]. To illustrate the underlying mechanism of the ATGL differential expression on LDs, we detected the possible triggers of the cAMP level and the PKA activity between hibernation and non-hibernation periods. We found that the activation of cAMP/PKA signaling during hibernation promoted ATGL recruitment onto the LD surface. CGI-58 is a coactivator protein for the full hydrolase activity of ATGL and deficiency of CGI-58 caused massive lipid accumulation in various tissues of mice [43]. Moreover, LD-associated proteins were recently proved to be involved in the CGI-58-mediated regulation of ATGL. In unstimulated adipose tissues, the interaction between perilipin-1 and CGI-58 prevents the combination of CGI-58 and ATGL, thus suppressing ATGL activation. In oxidative tissues, such as the liver and skeletal muscle, perilipin-5, rather than perilipin-1, regulates the activation of ATGL [17]. In the liver of the Chinese Soft-Shelled Turtle, the protein level of perilipin-5 decreased in the liver and cytosol, whereas the perilipin-5 content on LDs remained unchanged during hibernation. However, the phosphorylation of perilipin-5 on LDs increased. Meanwhile, the interaction between perilipin-5, CGI-58 and ATGL on the LDs increased during hibernation. These results indicated that phosphorylation of perilipin-5 on LDs induced ATGL enzyme activity through an interaction with CGI-58 and ATGL.

**Figure 6:**
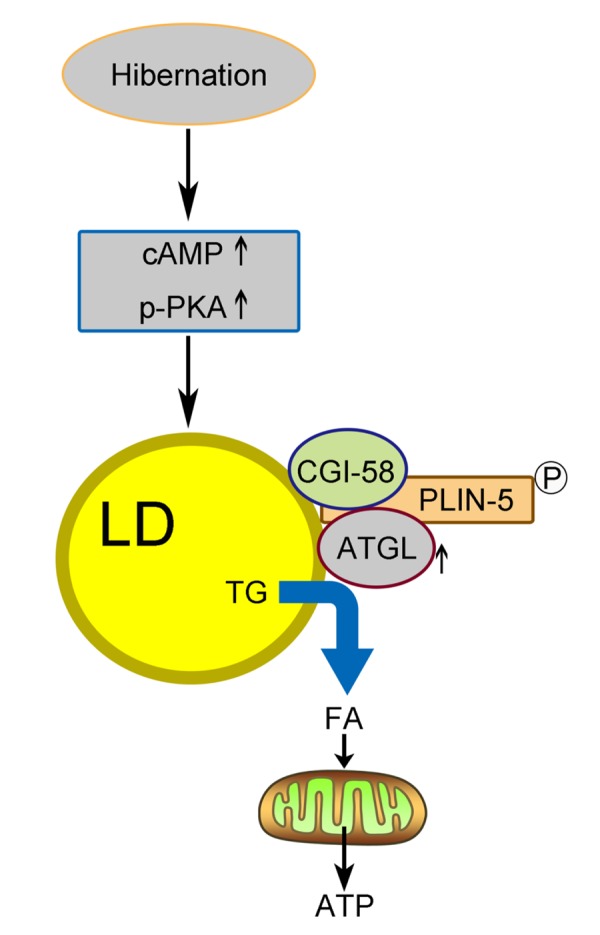
**Schematic diagram of LD breakdown during hibernation in the liver of Chinese Soft-Shelled Turtle (*Pelodiscus sinensis*).** Entering hibernation, activated cAMP/PKA signals in the liver recruit ATGL to the surface of the LDs. Meanwhile, phosphorylation of perilipin-5 on the LD surface promotes the CGI-58-mediated induction of ATGL via the interaction between CGI-58 and ATGL. Ultimately, FAs hydrolyzed from TGs stored in LDs are transported into mitochondria for β-oxidation, thereby maintaining energy hemostasis in the body.

In summary, we proposed a model (Figure 6) in which activated cAMP/PKA signaling during hibernation promotes the recruitment of ATGL onto the LD surface in the liver of Chinese Soft-Shelled Turtle. Meanwhile, the phosphorylation of perilipin-5 activates ATGL enzyme activity via the interaction of perilipin-5 with ATGL and CGI-58 when entering hibernation. This mechanism underlying hepatic LD degradation could partly explain how hibernating animals maintain hepatic homeostasis and survive during hibernation. Accordingly, perilipin-5 and CGI-58 could be potential therapeutic targets for age-related chronic liver disease (such as NAFLD).

## MATERIALS AND METHODS

### Animals

All procedures with turtles were conducted according to the Animal Research Institute Committee guidelines of Nanjing Agriculture University, China. Twenty 5-year old Chinese soft-shelled turtles were acquired from a wild breeding base in Nanjing, Southeast China (GPS coordinates N 32.050 E 118.783). The animals were collected in non-hibernation and hibernation periods. We feed the turtles in non-hibernation period and didn’t feed turtles in hibernation periods. After at least 24 hours, the turtles were anesthetized using intraperitoneal sodium pentobarbital (20 mg/kg) and killed by cervical bleeding. A portion of the liver was sampled immediately after death and prepared for transmission electron and light microscopy. The remaining part was kept under −70 °C for reverse transcription polymerase chain reaction (RT-PCR) and western blotting analysis. The Science and Technology Agency of Jiangsu Province and Nanjing Agricultural University Veterinary College approved the sampling procedures. The approval ID is SYXK (SU) 2010-0005. All efforts were made to minimize the animals’ suffering.

### Transmission electron microscopy (TEM)

The turtle liver was instantly sampled and cut into 1-mm^3^ blocks. The blocks were then immersed 2.5% glutaraldehyde overnight. Thereafter, the tissues were fixed in 1% osmium tetroxide and dehydrated in ascending concentrations of ethyl alcohol. Ethyl alcohol in the tissues was then substituted by acetone. Finally, the tissues were embedded in Epon812. Ultrathin slides were sectioned using an ultramicrotome and double stained with uranyl acetate and lead citrate. TEM images were taken under a Hitachi H-7650 instrument.

### Oil Red O staining

First, 6-μm-thick frozen slices were fixed in 10% neutral formalin for 10 minutes. After washing with distilled water for 2 minutes three times, the slides were immersed in Oil Red O solution for 10 minutes. Subsequently, the slides were washed with warm distilled water at 37 °C for 15 seconds. After counterstaining with hematoxylin for 2 minutes, the slides were rinsed with tap water for 60 seconds. Images were collected under a light microscope (Olympus DP73).

### Isolating LD and verifying the quality of isolated LDs

Liver LDs were isolated following an optimized version of the method of Liu et al. [23]. The purity of the LDs was validated through Nile red and Colloidal blue staining.

### Co-immunoprecipitation

Liver homogenates were lysed using co-immunoprecipitation (Co-IP) lysis buffer on ice for 30 minutes. The supernatants were collected after centrifugation at 12000 × *g* for 30 minutes at 4 °C. A small quantity of lysate was stored for western blot analysis. The immunoprecipitating antibodies and protein A/G beads were added into the remaining lysate, and then incubated at 4 °C overnight. After immunoprecipitation, the protein A/G beads were pelleted by centrifugation at 3000 × *g* for 5 minutes. The beads were washed with lysis buffer 3-4 times, and the proteins on the beads were extracted by adding protein loading buffer and boiling for 10 minutes. Subsequently, sodium dodecyl sulfate-polyacrylamide gel electrophoresis (SDS-PAGE) and western blotting were conducted for analysis.

### Western blotting

Samples of the livers from each group were homogenized using ice-cold radio immunoprecipitation assay (RIPA) buffer and centrifuged at 12000 × *g* for 10 minutes at 4 °C. Thereafter, the total protein concentration was determined using a BCA protein assay kit (Abcam, ab102536). Samples (40 µg of protein per lane) were subjected to electrophoresis on a 10% SDS-PAGE gel and then transferred onto PVDF membranes (Millipore, ISEQ00010). After nonspecific blocking in 5% nonfat milk, the membranes were incubated with antibodies recognizing cytochrome C, Cox IV, Tomm20, ATGL and perilipin 5, overnight at 4 °C. After washing with TBST, the membranes were incubated with peroxidase-linked goat anti-rabbit IgG (1:5000, Bioworld Technology Inc., BS13278) for 2 h. Following incubation, the bound antibodies were visualized using High-sig ECL Western Blotting Substrate (Tannon). Immunoreactive bands were quantiﬁed using Quantity One software (Bio-Rad Laboratories) (Supplementary Figure S1).

### RT-PCR

Total RNA of the turtle liver was extracted using the Trizol reagent. The purity of the extracted RNA was evaluated using the absorbance ratio at 260 and 280 nm (A260/A280) and the samples with ratios between 1.8 and 2.0 used in the next step. Total RNA was reverse transcribed into cDNA using the SuperScript First-Strand Synthesis System. Specific primers were designed using Primer3 Input (version 0.4.0) (Table 1) and the β-actin gene was used as an internal control.

**Table 1:** Primers used for RT-qPCR analysis in *P. sinensis.*

**Gene name**	**Sense primer (5'-3')**	**Antisense primer (5'-3')**
CPT1A	GCATGTTATTCCCACCTCCCT	TCCTGGGATACGGGAGGTATT
PPARA	ACAGGTGAACAGGATGTGGAA	TCTCTGCCATACACAGCGTT
APOB	CAGCCATCCAGGCATTGAGA	CTTGCTAAGATCGGACGGGG
FASN	CTCTCTGCCATCTCCCGAAC	GACTCCCATCTCCCTCCACT
ACACA	CATCCTTGGCTCTGTGTCTGA	TTACAAGGTGCAAGCCGGAC
SREBF1	CTTCGTGTGAAGACCAGCCT	GCTGTAGATGCTGTCCCTCG
ME1	ACGGCATGTGGAGGAATGAA	AGGCTTGACCCCTGACTCTT
ACADS	CCTTGGAAAGTGCTCGCTTG	ACAGTTGTGAAGCGTGTTCC
ACADM	GGCCTTGGCTCTTTTGAAGC	GCTCCAGGTTCTGTTACGCA
ACADVL	GGAGAGACAATCGCTGCCTT	GTCCTTCACAGGGGTCTTGG

### Statistical analysis

All data were presented as the means ± SE. The TEM pictures were imported into Image J software for statistical data analysis. The statistical analysis was performed using SPSS software version 14.0 with t tests and nonparametric tests. The data were considered statistically significant when P < 0.05.

## Supplementary Material

Supplementary Figure

## References

[1] Hall KE, Proctor DD, Fisher L, Rose S. American gastroenterological association future trends committee report: effects of aging of the population on gastroenterology practice, education, and research. Gastroenterology. 2005; 129:1305–38. 10.1053/j.gastro.2005.06.013 16230084

[2] Bertolotti M, Lonardo A, Mussi C, Baldelli E, Pellegrini E, Ballestri S, Romagnoli D, Loria P. Nonalcoholic fatty liver disease and aging: epidemiology to management. World J Gastroenterol. 2014; 20:14185–204. 10.3748/wjg.v20.i39.14185 25339806 PMC4202348

[3] Zhang L, Han XK, Li MY, Bao HJ, Chen QS. Spermiogenesis in soft-shelled turtle, Pelodiscus sinensis. Anat Rec (Hoboken). 2007; 290:1213–22. 10.1002/ar.20587 17724710

[4] Hu L, Li Q, Yang P, Gandahi JA, Arain TS, Le Y, Zhang Q, Liu T, Y Waqas M, Ahmad N, Liu Y, Chen Q. Expression of TLR2/4 on Epididymal Spermatozoa of the Chinese Soft-Shelled Turtle Pelodiscus sinensis During the Hibernation Season. Anat Rec (Hoboken). 2016; 299:1578–84. 10.1002/ar.23463 27532861

[5] White A, Handler P, Smith E and Stetten D, Jr. Principles of Biochemistry. 1954.

[6] Carey HV, Andrews MT, Martin SL. Mammalian hibernation: cellular and molecular responses to depressed metabolism and low temperature. Physiol Rev. 2003; 83:1153–81. 10.1152/physrev.00008.2003 14506303

[7] Blake BH. The annual cycle and fat storage in two populations of golden-mantled ground squirrels. J Mammal. 1972; 53:157–67. 10.2307/1378836 5016678

[8] Murphy DJ. The biogenesis and functions of lipid bodies in animals, plants and microorganisms. Prog Lipid Res. 2001; 40:325–438. 10.1016/S0163-7827(01)00013-3 11470496

[9] Zimmermann R, Strauss JG, Haemmerle G, Schoiswohl G, Birner-Gruenberger R, Riederer M, Lass A, Neuberger G, Eisenhaber F, Hermetter A, Zechner R. Fat mobilization in adipose tissue is promoted by adipose triglyceride lipase. Science. 2004; 306:1383–86. 10.1126/science.1100747 15550674

[10] Greenberg AS, Egan JJ, Wek SA, Garty NB, Blanchette-Mackie EJ, Londos C. Perilipin, a major hormonally regulated adipocyte-specific phosphoprotein associated with the periphery of lipid storage droplets. J Biol Chem. 1991; 266:11341–46. 2040638

[11] Jiang HP, Serrero G. Isolation and characterization of a full-length cDNA coding for an adipose differentiation-related protein. Proc Natl Acad Sci USA. 1992; 89:7856–60. 10.1073/pnas.89.17.7856 1518805 PMC49813

[12] Than NG, Sumegi B, Than GN, Kispal G, Bohn H. Cloning and sequence analysis of cDNAs encoding human placental tissue protein 17 (PP17) variants. Eur J Biochem. 1998; 258:752–57. 10.1046/j.1432-1327.1998.2580752.x 9874244

[13] Scherer PE, Bickel PE, Kotler M, Lodish HF. Cloning of cell-specific secreted and surface proteins by subtractive antibody screening. Nat Biotechnol. 1998; 16:581–86. 10.1038/nbt0698-581 9624692

[14] Wolins NE, Quaynor BK, Skinner JR, Tzekov A, Croce MA, Gropler MC, Varma V, Yao-Borengasser A, Rasouli N, Kern PA, Finck BN, Bickel PE. OXPAT/PAT-1 is a PPAR-induced lipid droplet protein that promotes fatty acid utilization. Diabetes. 2006; 55:3418–28. 10.2337/db06-0399 17130488

[15] Miyoshi H, Perfield JW 2nd, Souza SC, Shen WJ, Zhang HH, Stancheva ZS, Kraemer FB, Obin MS, Greenberg AS. Control of adipose triglyceride lipase action by serine 517 of perilipin A globally regulates protein kinase A-stimulated lipolysis in adipocytes. J Biol Chem. 2007; 282:996–1002. 10.1074/jbc.M605770200 17114792

[16] Granneman JG, Moore HP, Krishnamoorthy R, Rathod M. Perilipin controls lipolysis by regulating the interactions of AB-hydrolase containing 5 (Abhd5) and adipose triglyceride lipase (Atgl). J Biol Chem. 2009; 284:34538–44. 10.1074/jbc.M109.068478 19850935 PMC2787315

[17] Granneman JG, Moore HP, Mottillo EP, Zhu Z, Zhou L. Interactions of perilipin-5 (Plin5) with adipose triglyceride lipase. J Biol Chem. 2011; 286:5126–35. 10.1074/jbc.M110.180711 21148142 PMC3037624

[18] Fedorov VB, Goropashnaya AV, Tøien Ø, Stewart NC, Chang C, Wang H, Yan J, Showe LC, Showe MK, Barnes BM. Modulation of gene expression in heart and liver of hibernating black bears (Ursus americanus). BMC Genomics. 2011; 12:171. 10.1186/1471-2164-12-171 21453527 PMC3078891

[19] Xiao Y, Wu Y, Sun K, Wang H, Zhang B, Song S, Du Z, Jiang T, Shi L, Wang L, Lin A, Yue X, Li C, et al. Differential Expression of Hepatic Genes of the Greater Horseshoe Bat (Rhinolophus ferrumequinum) between the Summer Active and Winter Torpid States. PLoS One. 2015; 10:e0145702. 10.1371/journal.pone.0145702 26698122 PMC4689453

[20] Nelson CJ, Otis JP, Martin SL, Carey HV. Analysis of the hibernation cycle using LC-MS-based metabolomics in ground squirrel liver. Physiol Genomics. 2009; 37:43–51. 10.1152/physiolgenomics.90323.2008 19106184

[21] Zechner R, Zimmermann R, Eichmann TO, Kohlwein SD, Haemmerle G, Lass A, Madeo F. FAT SIGNALS--lipases and lipolysis in lipid metabolism and signaling. Cell Metab. 2012; 15:279–91. 10.1016/j.cmet.2011.12.018 22405066 PMC3314979

[22] Grasselli E, Voci A, Demori I, Vecchione G, Compalati AD, Gallo G, Goglia F, De Matteis R, Silvestri E, Vergani L. Triglyceride mobilization from lipid droplets sustains the anti-steatotic action of iodothyronines in cultured rat hepatocytes. Front Physiol. 2016; 6:418. 10.3389/fphys.2015.00418 26793120 PMC4709507

[23] Ding Y, Zhang S, Yang L, Na H, Zhang P, Zhang H, Wang Y, Chen Y, Yu J, Huo C, Xu S, Garaiova M, Cong Y, Liu P. Isolating lipid droplets from multiple species. Nat Protoc. 2013; 8:43–51. 10.1038/nprot.2012.142 23222457

[24] Duncan RE, Ahmadian M, Jaworski K, Sarkadi-Nagy E, Sul HS. Regulation of lipolysis in adipocytes. Annu Rev Nutr. 2007; 27:79–101. 10.1146/annurev.nutr.27.061406.093734 17313320 PMC2885771

[25] Kimmel AR, Sztalryd C. Perilipin 5, a lipid droplet protein adapted to mitochondrial energy utilization. Curr Opin Lipidol. 2014; 25:110–17. 10.1097/MOL.0000000000000057 24535284 PMC4517968

[26] Hibernation GF. Curr Biol. 2013; 23:R188–93. 10.1016/j.cub.2013.01.062 23473557

[27] Jessen N, Nielsen TS, Vendelbo MH, Viggers R, Støen OG, Evans A, Frøbert O. Pronounced expression of the lipolytic inhibitor G0/G1 Switch Gene 2 (G0S2) in adipose tissue from brown bears (Ursus arctos) prior to hibernation. Physiol Rep. 2016; 4:e12781. 10.14814/phy2.12781 27117803 PMC4848729

[28] Cotton CJ. Skeletal muscle mass and composition during mammalian hibernation. J Exp Biol. 2016; 219:226–34. 10.1242/jeb.125401 26792334

[29] McGee-Lawrence ME, Wojda SJ, Barlow LN, Drummer TD, Castillo AB, Kennedy O, Condon KW, Auger J, Black HL, Nelson OL, Robbins CT, Donahue SW. Grizzly bears (Ursus arctos horribilis) and black bears (Ursus americanus) prevent trabecular bone loss during disuse (hibernation). Bone. 2009; 45:1186–91. 10.1016/j.bone.2009.08.011 19703606 PMC2783552

[30] Chanon S, Chazarin B, Toubhans B, Durand C, Chery I, Robert M, Vieille-Marchiset A, Swenson JE, Zedrosser A, Evans AL, Brunberg S, Arnemo JM, Gauquelin-Koch G, et al. Proteolysis inhibition by hibernating bear serum leads to increased protein content in human muscle cells. Sci Rep. 2018; 8:5525. 10.1038/s41598-018-23891-5 29615761 PMC5883044

[31] Bonis A, Anderson L, Talhouarne G, Schueller E, Unke J, Krus C, Stokka J, Koepke A, Lehrer B, Schuh A, Andersen JJ, Cooper S. Cardiovascular resistance to thrombosis in 13-lined ground squirrels. J Comp Physiol B. 2019; 189:167–77. 10.1007/s00360-018-1186-x 30317383 PMC6335183

[32] Herinckx G, Hussain N, Opperdoes FR, Storey KB, Rider MH, Vertommen D. Changes in the phosphoproteome of brown adipose tissue during hibernation in the ground squirrel, *Ictidomys tridecemlineatus.* Physiol Genomics. 2017; 49:462–72. 10.1152/physiolgenomics.00038.2017 28698229 PMC5625268

[33] Carpentier AC, Blondin DP, Virtanen KA, Richard D, Haman F, Turcotte ÉE. Brown Adipose Tissue Energy Metabolism in Humans. Front Endocrinol (Lausanne). 2018; 9:447. 10.3389/fendo.2018.00447 30131768 PMC6090055

[34] Sommer F, Ståhlman M, Ilkayeva O, Arnemo JM, Kindberg J, Josefsson J, Newgard CB, Fröbert O, Bäckhed F. The gut microbiota modulates energy metabolism in the hibernating brown bear Ursus arctos. Cell Reports. 2016; 14:1655–61. 10.1016/j.celrep.2016.01.026 26854221

[35] Lass A, Zimmermann R, Oberer M, Zechner R. Lipolysis - a highly regulated multi-enzyme complex mediates the catabolism of cellular fat stores. Prog Lipid Res. 2011; 50:14–27. 10.1016/j.plipres.2010.10.004 21087632 PMC3031774

[36] Villena JA, Roy S, Sarkadi-Nagy E, Kim KH, Sul HS. Desnutrin, an adipocyte gene encoding a novel patatin domain-containing protein, is induced by fasting and glucocorticoids: ectopic expression of desnutrin increases triglyceride hydrolysis. J Biol Chem. 2004; 279:47066–75. 10.1074/jbc.M403855200 15337759

[37] Kershaw EE, Hamm JK, Verhagen LA, Peroni O, Katic M, Flier JS. Adipose triglyceride lipase: function, regulation by insulin, and comparison with adiponutrin. Diabetes. 2006; 55:148–57. 10.2337/diabetes.55.01.06.db05-0982 16380488 PMC2819178

[38] Jocken JW, Langin D, Smit E, Saris WH, Valle C, Hul GB, Holm C, Arner P, Blaak EE. Adipose triglyceride lipase and hormone-sensitive lipase protein expression is decreased in the obese insulin-resistant state. J Clin Endocrinol Metab. 2007; 92:2292–99. 10.1210/jc.2006-1318 17356053

[39] Kralisch S, Klein J, Lossner U, Bluher M, Paschke R, Stumvoll M, Fasshauer M. Isoproterenol, TNFalpha, and insulin downregulate adipose triglyceride lipase in 3T3-L1 adipocytes. Mol Cell Endocrinol. 2005; 240:43–49. 10.1016/j.mce.2005.06.002 16009485

[40] Chakrabarti P, English T, Shi J, Smas CM, Kandror KV. The mTOR complex 1 suppresses lipolysis, stimulates lipogenesis and promotes fat storage. Diabetes. 2010 10.2337/db09-1602 PMC2844824 20068142

[41] Liu LF, Purushotham A, Wendel AA, Koba K, DeIuliis J, Lee K, Belury MA. Regulation of adipose triglyceride lipase by rosiglitazone. Diabetes Obes Metab. 2009; 11:131–42. 10.1111/j.1463-1326.2008.00916.x 18643838 PMC3814028

[42] Chakrabarti P, Kandror KV. FoxO1 controls insulin-dependent adipose triglyceride lipase (ATGL) expression and lipolysis in adipocytes. J Biol Chem. 2009; 284:13296–300. 10.1074/jbc.C800241200 19297333 PMC2679428

[43] Radner FP, Streith IE, Schoiswohl G, Schweiger M, Kumari M, Eichmann TO, Rechberger G, Koefeler HC, Eder S, Schauer S, Theussl HC, Preiss-Landl K, Lass A, et al. Growth retardation, impaired triacylglycerol catabolism, hepatic steatosis, and lethal skin barrier defect in mice lacking comparative gene identification-58 (CGI-58). J Biol Chem. 2010; 285:7300–11. 10.1074/jbc.M109.081877 20023287 PMC2844178

