# Supplementary material for: Hepatic lipid droplet breakdown through lipolysis during hibernation in Chinese Soft-Shelled Turtle (Pelodiscus sinensis)
Source: Aging (Albany NY). 2019 Mar 29;11(7):1990–2002. doi: 10.18632/aging.101887 (PMC6503876; doi:10.18632/aging.101887)
Supplement: Supplementary Figure [file aging-11-101887-s001.pdf]

Supplementary Figure

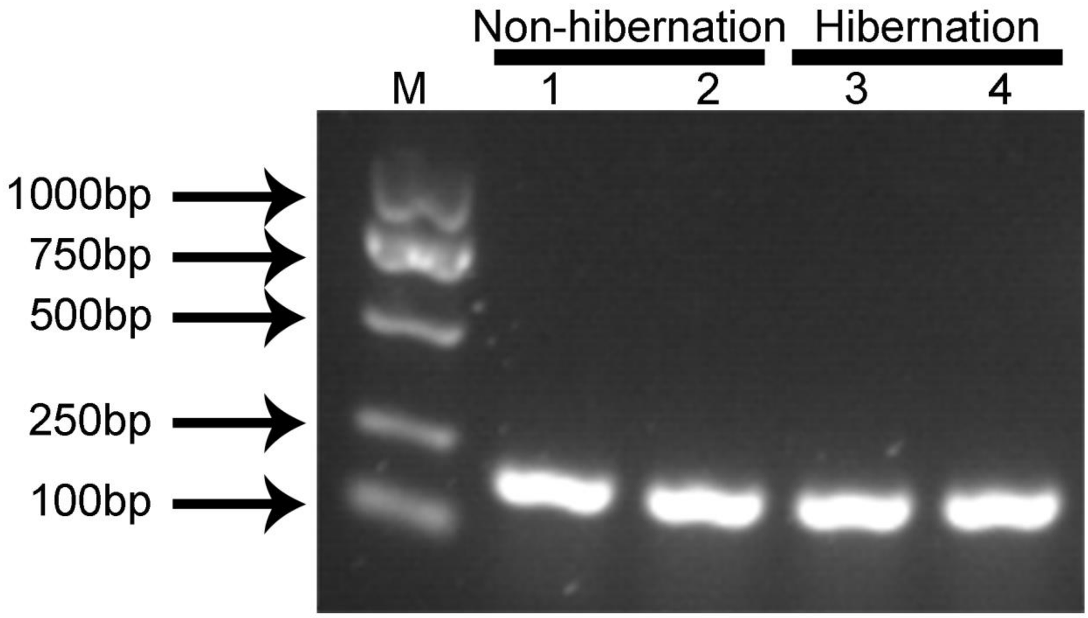

Supplementary Figure 1. Agarose gel electrophoresis (AGE) of β-Actin in the liver of Chinese Soft-Shelled Turtle during non-hibernation and hibernation periods. M: DNA marker DL 2000; Lane 1, 2: Non-hibernation; Lane 3, 4: Hibernation.
